# Ozone pollution: a persistent challenge in large cities like Mexico City, Los Angeles and Beijing

**DOI:** 10.14324/111.444/ucloe.3448

**Published:** 2025-10-30

**Authors:** Erik Velasco, Armando Retama, Luisa T. Molina

**Affiliations:** 1 Molina Center for Energy and the Environment, Boston, MA, USA

**Keywords:** ground-level ozone, ambient ozone, surface ozone, air quality, air quality management, environmental policy, atmospheric chemistry, urban air pollution

## Abstract

Over the past decade or so, many large cities around the world have made little to no progress in lowering ground-level ozone concentrations, despite significant reductions in key precursor pollutants directly emitted into the atmosphere. Ozone comes from complex chemical reactions in the air that make it difficult to control. Current control measures implemented in some cities have apparently reached their limit. While stricter emission regulations, improvements in technology and cleaner fuels have prevented a return to previous ozone levels, they have not reduced them further. They have triggered changes in the mixture of precursor species (i.e., nitrogen oxides and volatile organic compounds) due to differences in the extent to which their emissions have been reduced, emerging emission sources and the increasing relevance of emissions previously overlooked, such as those related to cooking and the use of household cleaning and personal care products. Similarly, as the contribution of typical emission sources (e.g., combustion of fossil fuels) has decreased, biogenic contributions have become more important, as well as the influence of regional and transboundary pollution. These changes have also responded to increasing urbanisation in the face of a changing climate that favours ozone production. There is no recipe that all cities can follow to tackle ambient ozone; however, it is necessary to review why ozone concentrations have not decreased in some cities and what they are doing about it in order to use it as a reference to update, improve and develop control measures tailored to local conditions, as actions implemented in one city may be ineffective or impractical in another. In such a context, this article examines the cases of three metropolitan areas: the Mexico City Metropolitan Area, the Los Angeles Basin and the Beijing–Tianjin–Hebei (*Jing–Jin–Ji*) region. These urban conglomerations, with different geography, meteorology, socioeconomic conditions and governance, have succeeded in reducing concentrations of many regulated pollutants to levels near or below air quality standards set to protect public health, but not for ozone. While these cities have robust and timely air quality management (i.e., air quality monitoring, emission inventories and air quality models), the current ozone challenge requires even greater efforts to understand the physical and chemical processes at the local and regional scales. This will enable informed actions that can adjust to changing environmental, social and economic scenarios, following a science-policy approach with a perspective of human rights and social justice.

## Introduction

Ground-level ozone (O_3_), also known as ambient or surface O_3_, plays a significant role in atmospheric chemistry by controlling the oxidation potential of the atmosphere [1]. However, at high concentrations, it poses a risk to public health [2], contributing to approximately 500,000 premature deaths annually on a global scale [3], with an estimated 93% occurring in urban areas [4]. It also damages forests and lowers crop yields [5,6], and acts as a short-lived climate forcer (SLCF, [7]). In such a context, urban, regional and even hemispheric O_3_ has been the subject of air quality management actions for several decades, with success in reducing mean concentrations and the number of critical high O_3_ episodes in many regions and cities [8,9]. However, progress has slowed recently, and even stalled in many large metropolitan areas like Mexico City [10,11], São Paulo [12], Los Angeles [13], New York [14], Rome [15], Madrid [16], London [17], Dresden and Leipzig [18], Moscow [19], Istanbul [20], Tehran [21], Beijing [22], Hong Kong [23], Tokyo [24] and Seoul [25], among many other cities around the world [26,27], where no significant reductions have been experienced since 10–20 years ago, despite large reductions in key pollutants directly emitted into the atmosphere, such as carbon monoxide (CO), sulfur dioxide (SO_2_), particulate matter (PM), especially those smaller than 10 μm (PM_10_), nitrogen oxides (NO_X_ = NO + NO_2_, nitric oxide + nitrogen dioxide) and volatile organic compounds (VOCs). On the contrary, in cities such as Jakarta, where emissions of these pollutants of primary origin continue to increase, O_3_ concentrations, ironically, show a downward trend [28].

On a global scale, NO_X_, VOCs, CO and methane (CH_4_) drive O_3_ production. In urbanised areas and downwind regions, O_3_ formation is mostly driven by local NO_X_ and VOC emissions [29]. VOCs and NO_X_ are emitted into the atmosphere by both anthropogenic and natural emission sources. Consumption of fossil fuels for transporting people and goods, industry and power generation, and household activities are main emission sources of both precursor pollutants [30–32]. The use of biomass for domestic heating and cooking also emits VOCs and NO_X_ [33]. The so-called volatile chemical products (VCPs = coatings, inks, adhesives, personal care products, cleaning agents and pesticides) have gained relevance as anthropogenic VOC emission sources [34]. Vegetation is also a major source of reactive VOCs, especially during the growing season [35]. At a regional scale, wildfires are important sources of VOCs and NO_X_ [36], and soil microbial activity of NO_X_ [37,38].

The ratio between VOCs and NO_X_ determines the O_3_ production regime and sheds light on the required emission control measures to reduce ambient O_3_ concentrations. Under high VOC and low NO_X_ concentrations (i.e., NO_X_-limited regime), O_3_ production increases with increasing NO_X_. On the contrary, if NO_X_ concentrations are high relative to VOCs (i.e., VOC-limited regime), the opposite effect is observed: increasing VOCs or reducing NO_X_ enhances O_3_ production [39]. In other words, in the presence of a NO_X_-limited regime, reductions in NO_X_ emissions are most beneficial, whereas in the presence of a VOC-limited regime, reductions in VOCs are most effective. Changes in the temporal and spatial distributions of VOCs and NO_X_ ratios, resulting from changes in the abundance of both precursor species due to stricter emission regulations, advanced technologies, emerging emission sources, growing urbanisation and economic expansion, alongside a changing climate that favours O_3_ production and increases in background O_3_ levels caused by transboundary pollution, are preventing further reductions in O_3_ concentrations. Furthermore, it has been noted that reductions in particulate pollution may result in increased O_3_ levels if VOC and NO_X_ are not reduced simultaneously to a sufficient extent [40]. Reducing particles increases the availability of hydroperoxyl radicals, which are essential for O_3_ production. Decreasing particle loading can also result in other heterogenous reactions, variations in photolysis rates and changes in local micrometeorology that may increase O_3_ levels. All of these factors combined are making it increasingly difficult to meet concentration thresholds assigned as air quality standards to protect public health [41], as well as the World Health Organization (WHO) air quality guidelines (AQG) recommended for the same purpose [42].

**Figure 1:**
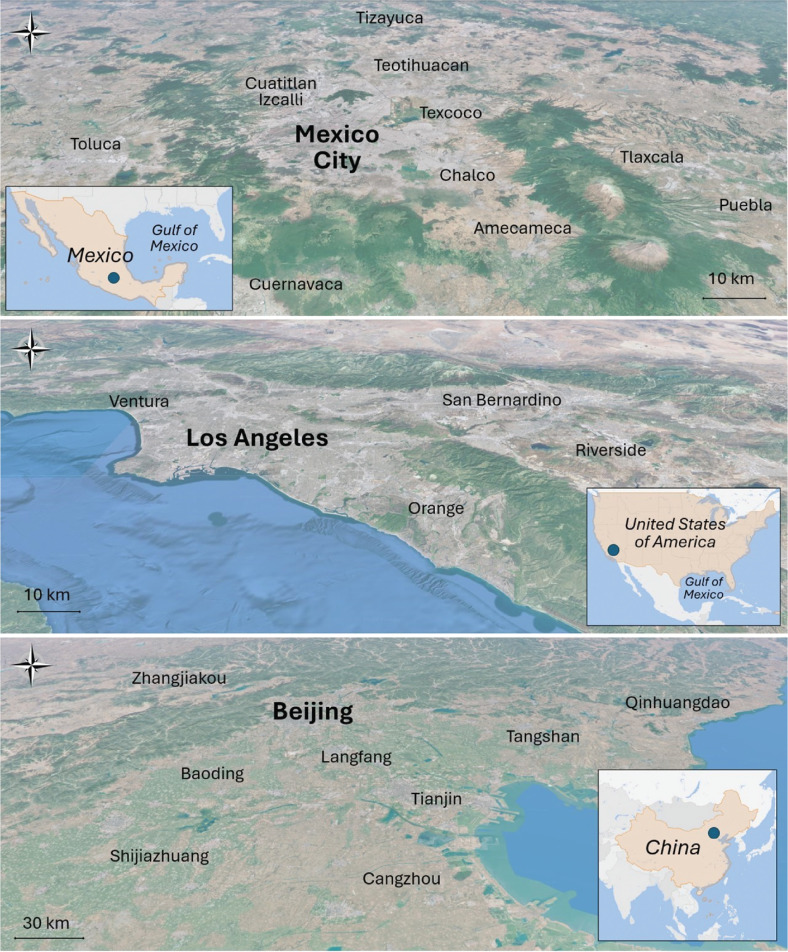
Geographical features of the three metropolitan areas examined in this article: (a) MCMA, (b) LA Basin and (c) *Jing–Jin–Ji*. Maps source: Google Earth.

In such a context, this article examines three representative cases: the Mexico City Metropolitan Area (MCMA), the Los Angeles Basin (LA Basin), and the Beijing–Tianjin–Hebei Region (BTH, also known as *Jing*–*Jin*–*Ji*, based on the Chinese abbreviations for Beijing (*Jing*), Tianjin (*Jin*), and Hebei (*Ji*)) to illustrate the challenges currently facing large metropolitan areas in controlling ground-level O_3_ pollution. Each of these urban conglomerations presents distinctive geographical, climatological, social and economic characteristics, as well as particular governance issues that appear to impede further progress in reducing ambient O_3_ concentrations. Figure 1 shows the geographical features of these three metropolitan areas.

**Figure 2:**
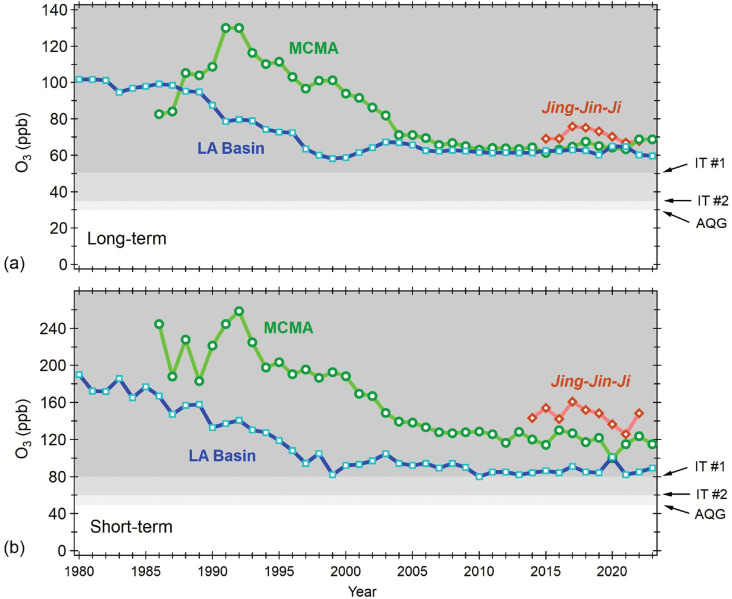
Annual long- and short-term exposure O_3_ trends at the MCMA, LA Basin and *Jing–Jin–Ji* regions following the AQG and ITs proposed by the WHO [42]. The IT #1 and #2, and AQG for long-term exposure of 50, 35 and 30 ppb (at 20°C and 1013 hPa), respectively, correspond to peak-season exposure, defined as the six consecutive months of the year exhibiting the highest 6-month running-average 8-h mean concentration. Similarly, the IT #1 and #2, and AQG for short-term exposure of 80, 60 and 50 ppb, respectively, are defined as the 99th percentile of the annual distribution of daily maximum 8-h mean concentration. Data were obtained from the Mexico City’s Air Quality Monitoring System (https://www.aire.cdmx.gob.mx/), the United States’ Environmental Protection Agency (https://www.epa.gov/outdoor-air-quality-data), and the TOAR Database for the case of the *Jing–Jin–Ji* region (https://doi.org/10.5281/zenodo.10911197).

These metropolises have attained concentrations of other key pollutants below or close to air quality standards, but O_3_ remains a challenge, even though stricter emission regulations, improved technology and cleaner fuels have been effective in reducing emissions of precursor pollutants. They have not yet achieved the Interim Target (IT) #1 of 50 and 80 ppb proposed by WHO for both long- and short-term exposure, respectively (see Fig. 2).

These three case studies do not seek to cover all the causes leading to high ambient O_3_ concentrations in every city in the world, but they do aim to show the complexity of controlling O_3_ pollution under a changing climate and growing urbanisation. The material presented in this article is intended to serve as a reference for future science-based dialogues involving policymakers, air quality managers, practitioners, scientific community and the non-governmental organisations in searching for sustained policies to eliminate the threat posed by O_3_ in large cities. The experience achieved in these metropolises shall be taken as a reference, especially in urban areas where rapid economic growth and urbanisation demand urgent actions to improve the quality of the air that their citizens breathe.

Each case study is presented separately, beginning with a brief overview of their individual O_3_ problem, followed by a description of their geographical and topographical settings, climatological characteristics and urbanisation patterns. Ozone data collected by local air quality monitoring networks, as well as relevant scientific literature, are used to assess historical trends and progress or stagnation in reducing O_3_ concentrations.

The health and economic impacts are then presented, along with a review of the main interventions, regulations and policies implemented so far, concluding with the challenges that they face ahead. Table 1 shows general features of these three metropolitan areas to put their current situation in context. It is worth noting that the *Jing–Jin–Ji* region is far larger than the MCMA and the LA Basin, with a population five times greater. This is due to China’s approach to managing air quality. The article concludes by summarising key lessons learned from the three cases, analysing similarities and differences and presenting a set of recommendations based on their experiences.

**Table 1.** Characteristics of the three metropolitan areas examined in the present work relevant to ground-level O_3_ pollution

	MCMA	LA Basin	*Jing–Jin–Ji* region
Population	21.8 million (2024)	18.3 million (2023)	110 million (2024)
Total area (km^2^)	7866	5908	212,000
Population density (inhabitants per km^2^)	2770	3100	520
GDP per capita in US dollars	15,700 (2022)	88,000 (2023)	13,430 (2023)
Topography	High altitude basin (2240 masl) surrounded by mountains and volcanoes on three sides	Coastal plain surrounded by mountain ranges to the north, east, and southeast	Cluster of cities along the coast of the Bohai Sea
Climate zone	Subtropical highland climate	Borderline Mediterranean and semi-arid climate	Monsoon-influenced humid continental climate
Vehicular fleet (million vehicles)	5.65	15.67 (2024, https://www.dmv.ca.gov)	26.7 (2020)
O_3_ air quality standards	65 ppb (8-h average)	70 ppb (8-h average)	80 ppb (8-h average), 100 ppb (1-h average)
Number of air quality monitoring stations	46	38	97
O_3_-attributable mortality cases	580 [43]	810 [4]	4282 in 2019 [44]
Agency responsible for regulating air pollution	Secretary of the Environment of Mexico City (SEDEMA, Spanish acronym): https://www.aire.cdmx.gob.mx/	California Air Resources Board (CARB): https://ww2.arb.ca.gov/	Ministry of Ecology and Environment of the People’s Republic of China: https://www.mee.gov.cn/

The historical running-average 8-h concentrations used to elaborate time series and determine the fraction of days exceeding current air quality standards are made available to the public by the environmental authorities in the MCMA and the LA Basin, whereas in the *Jing–Jin–Ji* region, they can be accessed through the Tropospheric Ozone Assessment Report (TOAR) Database. Hourly records from each monitoring station in the metropolitan areas were used to calculate the metrics presented in the figures throughout the article.

The article focuses on the role of O_3_ as an airborne pollutant rather than as an SLCF. Ozone acts as a direct climate forcer but has a relatively short atmospheric lifetime, ranging from a few hours to weeks, in contrast to carbon dioxide (CO_2_), which can last for centuries or even thousands of years [45]. Ambient, surface or ground-level O_3_ should not be confused with tropospheric O_3_. The three former terms are equivalent and only consider the abundance of O_3_ within the boundary layer and not over the entire troposphere. The thermal structure of the atmosphere creates a distinct vertical O_3_ distribution. Ozone in the upper troposphere is important for climate change but not for air quality. On the contrary, it is true that reducing emissions of combustion-related O_3_ precursors does cut CO_2_ emissions; thus, air quality and climate change programmes should be coordinated to provide synergistic benefits. However, this must be done with caution because emission reductions may not always result in lower O_3_ production, as will be seen later. Also, keep in mind that climate change benefits may be limited and temporary, as O_3_ impact depends on seasonal variability and latitude. This is why control strategies aimed at reducing urban O_3_ must prioritise air quality.

## Mexico City Metropolitan Area (MCMA)

Despite stringent control measures in place, ambient O_3_ pollution remains a threat to public health in Mexico City and its surrounding metropolitan area. A significant reduction in O_3_ concentrations was achieved towards the end of the last century and at the beginning of the current century, but since c. 2010, concentrations have stalled and may have started to increase again [11]. Current O_3_ concentrations exceed air quality standards in Mexico on more than half of the days, with concentrations high enough to trigger 3–5 air quality contingencies each year. This has a hefty burden on public health: 2.6 premature deaths and 42 disability-adjusted life years (DALYs) are predicted per 100,000 inhabitants [43]. The control measures implemented about 20 years ago were based on scientific information and demonstrated that large and complex cities can improve air quality without hindering economic growth. Indeed, those measures have kept O_3_ pollution from rising steeply again, but a different mix of precursor pollutants as a result of the implementation of advanced technology and new regulatory measures, emergent emission sources and a growing urban sprawl with a different landscape under a changing climate have limited their effectiveness and prevented further reductions.

### General characteristics

With more than 21 million inhabitants, the MCMA is the most populous metropolitan area in North America. It expands over 7866 km^2^, occupying the entire area of Mexico City and 60 neighbouring municipalities of two surrounding states. However, five metropolitan areas, also located in the central region of Mexico, have experienced a rapid expansion, producing a contiguous urban complex known as the Mexico Megalopolis, which covers nearly 100,000 km^2^ with a population of 41.5 million inhabitants [46].

The MCMA is located within a semi-closed basin on the central Mexican Plateau at an elevation of 2240 m above sea level in the tropics (20° N). It has a highland subtropical climate with a well-defined rainy season and two dry seasons, one warm and one cool. The warm dry season, which lasts from March until the end of May, is characterised by high-pressure systems with clear skies and strong solar radiation, which boost photochemistry and result in an increase in O_3_ production [10,11]. O_3_ pollution is less intense during the other two seasons, but high O_3_ concentrations cannot be ruled out, especially on days with low cloud cover and weak winds. The high elevation and the basin–mountain circulation ventilate the city effectively, while the heat absorbed by the built-up surface creates unstable atmospheric conditions and a relatively high boundary layer, so there is relatively little daily accumulation of pollutants; however, due to its subtropical latitude and high altitude, shortwave solar radiation is intense and enhances photochemistry, and severe O_3_ episodes can occur throughout the year.

### Ozone trends

**Figure 3:**
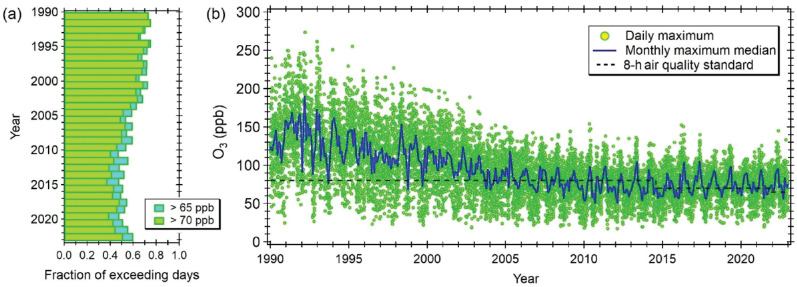
Daily 8-h average maximum O_3_ concentrations. (a) Fraction of days exceeding the current local 8-h air quality standard of 65 ppb and the 70 ppb standard that was in effect from July 2014 to October 2021. (b) Time series of daily 8-h average maximum concentrations. Green markers indicate the maximum concentration reported by any air quality monitoring station across the metropolitan area. The blue line is the monthly median of the daily maximum concentrations. Data were obtained from the Mexico City’s Air Quality Monitoring System (SIMAT, Spanish acronym; https://www.aire.cdmx.gob.mx/).

In the early 1990s, Mexico City acquired the infamous title of the world’s most polluted city [47], when the newly established air quality monitoring network revealed O_3_ concentrations above 300 ppb for 40–50 days annually. Over the next 15 years, annual average concentrations decreased by about 65%. There was a steady decline in O_3_ peak concentrations, with most days falling below the current hourly threshold of 155 ppb before an air quality contingency is activated (see Fig. 3). Nonetheless, nowadays 60–70% of the days still experience peaks above the 1-h average concentration (90 ppb), and 50–55% exceed the 8-h moving average concentration (65 ppb) deemed unhealthy by Mexican legislation (i.e., air quality standards).

No improvement in O_3_ concentrations has been observed since around 2010, and the evidence suggests a possible rebound back to elevated concentrations. Molina et al. [11] determined a mean increase of 0.39 ppb per year during the period 2010–2022. This implies an increase of ∼5 ppb in 12 years. Albeit with periodic reductions in the contingency threshold to adjust for management progress, the O_3_ alarm has rung at least once every year since 2016.

The ambient concentrations of other key pollutants, such as SO_2_ and CO, have decreased by more than 90% since 1990 and now are below current air quality standards. Concentrations of NO_X_ and PM_10_ have also decreased substantially by around 50% and 70%, respectively, but remain above respective air quality standards. Similar to O_3_, concentrations of particles smaller than 2.5 μm (PM_2.5_) have not decreased in 15 years.

Environmental authorities attribute the growing trend in O_3_ concentration to changes in atmospheric chemistry produced by the differences in the extent to which emissions of NO_X_ and VOCs have been reduced [48]. Indeed, changes in the mix of precursor pollutants responding to improved emission control regulations, technological advances and emerging emission sources may enhance the production of O_3_ rather than slow it [49]. It is also necessary to consider the expansion of the urban sprawl and changes in the urban morphology (e.g., increasingly taller buildings), which alter the production of turbulence and wind circulation within the basin, with a direct impact on the city’s ventilation and pollutants’ dispersion. Regional air pollution is becoming increasingly important. Emissions from the other five major cities within the Mexico Megalopolis may now have a greater impact on the MCMA’s air quality and vice versa [50]. Finally, changes in regional meteorology favouring clearer skies [51], and meteorological anomalies associated with climate change, such as heat waves and upper tropospheric troughs triggered by jet streams reaching lower latitudes, can produce high O_3_ pollution episodes [52].

### Health and economic impacts

In terms of public health, the progress that was achieved up to 15 years ago is undeniable. For example, Rojas-Bracho et al. [53] determined that O_3_ reductions in Mexico City between 1990 and 2015 prevented approximately 4100 premature deaths. Similarly, by reducing 10% of the 1-h O_3_ peak concentrations, authorities estimated that 124 annual premature deaths could be prevented [54].

The 14 measures outlined in the current Air Quality Improvement Program (*Programa Para Mejorar la Calidad del Aire, ProAire ZMVM 2021–2030*) seek to cut VOC and NO_X_ emissions by at least 26% and 40%, respectively, and reduce the annual O_3_ concentrations by 2% (∼3 ppb) and the maximum hourly concentration by 7% (∼31 ppb) [48]. Considering the total burden of pollutants, these measures are expected to prevent 6000 annual premature deaths by 2030, which is equivalent to an estimated economic value of US$5880 million or about 2.3% of the current gross domestic product (GDP) in the MCMA. By then, these economic benefits would have offset the implementation costs of the entire programme by more than 40% [48].

### Main interventions

Efforts to reduce air pollution began in the 1970s, but it was not until 1993 that the Mexican Government enacted the first air quality standards to protect public health. Since then, air quality has been the subject of political action. At the time, the federal government enacted the General Law of Ecological Equilibrium and Environmental Protection, which set clear jurisdictional responsibilities for addressing air pollution at the federal, state and local levels. At the same time, a metropolitan environmental commission that years later evolved into the Environmental Commission of the Megalopolis (*Comisión Ambiental de la Megalópolis*, CAMe) was established to coordinate the environmental actions between the Federal Government, and the local governments of the states that make up the MCMA [55].

Plans to reduce air pollution are periodically revised and updated. The Air Quality Improvement Program (*ProAire*) acts as a preventive and corrective instrument to improve air quality and protect public health, as well as to comply with the applicable legal framework [48]. The first air quality management programme was implemented in 1990 and has been updated five times. The strategies shaped in these programmes respond to the integration of air quality information obtained by authorities and academia to understand the origin, transformation and fate of air pollution, as well as to evaluate its impact on public health and economic growth. A series of studies conducted as part of three intensive air quality field campaigns between 1997 and 2006 were fundamental in integrating the air quality management programmes and succeeded in reducing pollution levels 15–20 years ago [56–58].

Despite notable differences in institutional capacity, financial resources and technical expertise, the MCMA drew lessons from the LA Basin, which began its air cleanup efforts two decades earlier. Similar strategies and emission control technologies were initially implemented [55].

The policies proposed in the current *ProAire* centre on improving vehicle technology and fuel quality, lowering emissions in industries and the commercial sector, increasing public transportation, improving urban planning and encouraging environmental education and research [48]. They are backed by an integrated financial strategy aiming at achieving sustainability by developing mechanisms that produce economic benefits, offset programme investment expenses, and become self-sustaining through the benefits of breathing clean air. It combines investments, employment and air quality in an effort to direct human and financial resources into initiatives involving the private sector, academia and society.

### Challenges ahead

Despite significant progress in improving air quality from the late 1990s to around 2010, the MCMA is on the verge of returning to previous O_3_ pollution levels due to several contributing factors, as described. The most prominent of these factors appears to be urban expansion. The surrounding municipalities are of the greatest concern, as urban sprawl has grown with little or no planning across them. Except for the MCMA, there is limited air quality monitoring and air pollution studies in the entities that comprise the Mexico Megalopolis. This makes it difficult to assess the regional air quality and its impact on the MCMA. The different administrative and legislative jurisdictions of these entities, and the limited available financial resources, have created an ongoing challenge that does not contribute to solving the air pollution problem.

New financial schemes and political will are needed to ensure robust air quality management across the region to enable timely and informed action. Local governments and federal agencies must foster a spirit of openness and collaboration and be receptive to independent science-policy advice in order to find long-term sustained solutions. In this context, a vigorous and active academic community is needed. Scientists must fill gaps in knowledge and influence policymakers. Policies must be in line with the scientific knowledge and evidence collected locally. The current challenge posed by persistently high O_3_ concentrations demands stakeholders who are fully committed to society and specialists with knowledge in atmospheric sciences.

## LA Basin

The LA Basin has been a leader during the last 50 years in efforts to improve air quality. However, because of its immense vehicular traffic, large industrial presence and geographical features, improving air quality has been challenging. Significant reductions were achieved over the past century due to extensive control measures, including strict regulations and market-based mechanisms to reduce vehicular and industrial emissions, improved fossil fuels, financial incentives for low-emission technology adoption, and more recently, strategies targeting emissions from households. Nevertheless, despite ongoing decreases in O_3_ precursor pollutants, negligible reductions have been observed this century. Current O_3_ concentrations exceed the local air quality standard on almost 25% of the days each year, especially during the summer. This has a significant impact on public health, with 5.4 premature deaths [4] and 79 DALYs predicted per 100,000 inhabitants [43]. Changes in emission profiles and photochemistry, alongside background O_3_ concentrations due to transboundary pollution, are the main culprits. Nowadays, non-traditional emission sources, such as the use of cleaning and personal care products and cooking, as well as biogenic emissions, play a significant role in O_3_ production. In addition, recent studies have found that current O_3_ pollution is altered by climate change phenomena, such as heat waves, drought and wildfires [36,59,60].

### General characteristics

The LA Basin covers five counties (Los Angeles, Orange, Riverside, San Bernardino and Ventura), being the second largest urban area in the United States and California’s largest metropolitan region. It is home to approximately 18 million people, over 40% of California’s population. It is also home to over 12 million passenger vehicles that, together with the commercial vehicles on the roads, travel over 230,000 million km per year. Emissions from these vehicles, along with those from ships, ports, rail yards and airports, combined with stationary sources such as refineries and power plants, and emissions from households, all contribute to O_3_ air pollution [13,61]. It is situated in the South Coast Air Basin, bordered by the Pacific Ocean on the west and mountains on the other three sides. The transport of air pollutants into and out of the basin is determined by factors such as sea breezes and topography-driven winds. The interplay of these factors frequently causes air masses to circulate back and forth daily [62]. Consequently, air masses can become trapped within the mountainous coastal region for several days, allowing for extensive photochemical processing and formation of O_3_. Ozone concentrations are highest in the spring through early fall.

### Ozone trends

**Figure 4:**
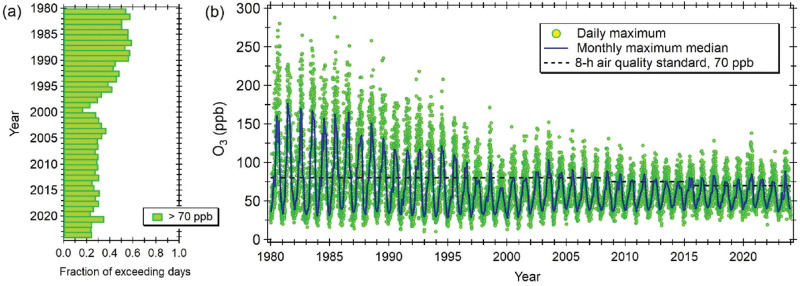
Daily 8-h average maximum O_3_ concentrations. (a) Fraction of days exceeding the current local 8-h air quality standard of 70 ppb. (b) Time series of daily 8-h average maximum concentrations. Green markers indicate the maximum concentration reported by any air quality monitoring station across the LA Basin. The blue line is the monthly median of the daily maximum concentrations. Data were obtained from the United States’ Environmental Protection Agency (EPA, https://www.epa.gov/outdoor-air-quality-data).

The LA Basin is an example of an urban region with long-standing O_3_ pollution. Substantial reductions in O_3_ concentrations were achieved during the last three decades of the 20th century thanks to extensive pollution control measures [63]. In the early 1980s, daily 8-h average peak concentrations over 200 ppb were recorded for 20–30 days each year; the last day exceeding this threshold was in 1994. Peak 8-h average concentrations in the late 1990s revolved around 120–150 ppb; in 2000, only two days exceeded 120 ppb. By 2010, the number of days above 100 ppb had dropped to a single digit, albeit 2023 and 2024 experienced over 10 days with higher peak concentrations. No further reductions have been achieved during the last two decades, and even small increases have started to be observed, despite important emission reductions of precursor pollutants, NO_X_ and VOCs, of anthropogenic origin [13]. Nowadays, 25–30% of the days each year, the majority during the summer, exceed the 8-h air quality standard of 70 ppb for health protection (see Fig. 4).

Continuous efforts to decrease emissions from vehicular traffic and stationary sources have enabled NO_X_ emission reductions of more than 50% since the late 1980s. Similarly, total VOC emissions have decreased by 60–70% [13]. Control measures have successfully reduced traffic emissions by a factor of 4–5. The contributions of so-called VCPs, associated with household and commercial activities previously overlooked, have gained relevance, despite a two-fold decline [13]. Studies suggest that evaporative emissions of these products and other non-traditional anthropogenic sources, such as cooking, are currently the largest source of anthropogenic VOCs with significant impacts on O_3_ production [64,65]. As anthropogenic emissions have dropped, biogenic contributions have increased. According to recent studies, vegetation is likely responsible for 50–60% of current summer O_3_ concentrations [66]. Biogenic emissions are expected to increase further due to a clear trend of rising temperatures.

Reducing the emissions of one or both O_3_ precursors (i.e., VOCs and NO_X_) would not necessarily inhibit O_3_ production. Historically, the LA Basin has been under a VOC-limited regime due to an overabundance of NO_X_, but about 20 years ago, it entered into a transitional state, moving towards an NO_X_-limited regime as VOC reductions have been outpaced by NO_X_ reductions, and no further O_3_ reduction has been achieved [67]. Recent studies have suggested that drastic reductions in NO_X_ emissions, larger than those experienced during the COVID-19 pandemic (20–30%), are needed to reduce O_3_ concentrations; otherwise, temporal O_3_ increases could be expected [67–69]. However, meteorological variability triggered by climate change may obscure the effectiveness of such reductions. Heat waves and wildfires enhance O_3_ pollution [36,60], while droughts may reduce O_3_ production by decreasing biogenic emissions [59].

In addition to locally produced O_3_, polluted plumes from East Asia crossing the Pacific Ocean and entering California complicate efforts to reduce O_3_ concentrations in the LA Basin. These plumes add on average 45 ppb, but can occasionally add up to 60 ppb, which is comparable to concentrations resulting from local production [70].

### Health and economic impacts

Malashock et al. [4] projected 5.4 premature deaths per 100,000 inhabitants, or around 810 total deaths, from O_3_ exposure in the LA Basin for 2019. The Institute for Health Metrics and Evaluation (IHME) estimated that O_3_ pollution caused 79 years of life lost from premature death and living in poor health (DALYs) for the entire state of California in 2024 [43]. According to IHME, the number of premature deaths has not changed since the 1990s, while the number of DALYs has decreased 15–20%.

Stewart et al. [71] evaluated the public health and financial benefits associated with less O_3_ pollution in the LA Basin by reducing NO_X_ and VOC emissions by 61% and 32%, respectively, using 2008 as a reference year. They found that 204 premature deaths, 660 emergency hospital admissions and 236,000 cases of acute respiratory symptoms could be avoided, with an estimated economic value of US$2645 million, or about 0.22% of the local GDP adjusted to current purchasing power.

### Main interventions

The first recognised air quality episode in Los Angeles occurred in the summer of 1943 [72]. However, it was not until 1967 that the California Air Resources Board (CARB) was established. CARB’s mission is to promote and protect public health, welfare and ecological resources through the effective and efficient reduction of air pollutants while recognising and considering the economic effects [72]. All air quality programmes are founded on scientific research, seeking out the pollutants’ origin and their consequences on human health and the environment. For such an endeavour, CARB counts on a robust air quality monitoring network with stations strategically located to assess the pollutants spatial distribution within and outside metropolitan areas [72]. Air quality data is made available to the public in real time and used in combination with emission inventories and air quality modelling forecasts to provide accurate and timely advice to people. CARB is in charge of developing and amending statewide regulations, as well as monitoring regulatory action. CARB develops and adopts regulations through a process that allows for public input by encouraging the involvement of stakeholders and citizens through board hearings and workshops. Parrish et al. [61] provides a summary of CARB’s evolution and main tasks to overview air quality throughout California.

A wide array of emission control technologies and regulations have been developed through years of scientific and engineering research. Efforts are focused on reducing anthropogenic emissions from all major sources by implementing strict regulations, introducing cleaner fuels and promoting the use of advanced technology. Some of the most significant actions have been the following:

1. Mobile sources: rigorous emission regulations in passenger cars, motorcycles, commercial trucks, public buses and off-road equipment such as lawn mowers have been implemented. Catalytic converters were introduced for the first time in 1975, and the installation of diesel exhaust filters in heavy-duty trucks became mandatory in 2008. Every 5 years, CARB has to review and update strategies on mobile emissions to meet California’s clean air and climate change targets. For such an endeavour, CARB uses scenario planning tools to model levels of clean technologies needed and identifies actions to reduce vehicle distance travelled and high-level concepts for each mobile source sector that could be further developed into regulations and other programmes to achieve these levels of cleaner technology [73]. The next revision was scheduled to be published in late 2025, but at the time of writing, its development was on hold due to staff cuts and administrative issues at the federal level.
2. Goods movement sources: railroads, oceangoing vessels, commercial harbour craft, cargo handling equipment, drayage trucks and transport refrigeration have gone through various regulations to reduce their emissions. For example, through the continuously revised Advanced Clean Fleets Regulation [72], CARB has attained significant emission reductions from medium- and heavy-duty vehicles [74]. The use of clean fuels by ships approaching California’s coast was adopted in 2008, and it has been effective in reducing emissions from the shipping sector [75].
3. Cleaner fuels: various diesel and gasoline quality standards have been adopted over the years [72], including low-carbon standards to reduce carbon intensity [76].
4. Area sources: regulations have been imposed on emission sources that individually emit small quantities of pollutants but collectively are significant. Over 100 categories of consumer products have been standardised, resulting in nearly a 50% reduction in emissions since 1990 [72].
5. Industrial sources, including power plants, refineries, manufacturing facilities and small stationary sources such as gasoline service stations, dry cleaners, bakeries, workshops, etc., have been equipped with the best available control technology [72].

Simultaneously, CARB and the South Coast Air Quality Management District (SCAQMD) have used strategies that foster technological progress and self-regulation of emissions to implement such control measures. Three key strategies have been the following:

1. The Regional Clean Air Incentives Markets (RECLAIM) Program reduces industrial NO_X_ and SO_X_ emissions by using market-based mechanisms [77].
2. The Carl Moyer Memorial Air Quality Standards Attainment Program provides financial incentives for low-emission technology adoption. It provides grant funding for cleaner-than-required engines, equipment and other air pollution sources [72].
3. The Consumer Products Regulatory Program seeks to lower VOC emissions from household and personal care products by working with industry and other stakeholders to achieve feasible VOC emission reductions without eliminating such products from the market [72].

### Challenges ahead

California is taking bold steps toward near-zero carbon emissions under a circular economy model that will undoubtedly contribute to reducing emissions of both greenhouse gases and O_3_ precursors, such as initiatives to phase out internal combustion engines in light-duty and heavy-duty vehicles [72]. However, at the time of writing this article in September 2025, the shifting political landscape at the federal level was holding back the implementation of environmental initiatives like this. A number of federal policies had already been proposed to roll back some environmental regulations and reduce research funding, notably related to climate change initiatives and environmental justice.

The federal waiver granted to the State of California by the Environmental Protection Agency (EPA) under the Clean Air Act to request the ability to enact more stringent emissions standards for new motor vehicles compared to federal regulations had not been revoked [78]. However, multiple challenges were underway regarding California’s waivers, including US Supreme Court cases and Congressional Review Act resolutions, creating uncertainty in the regulatory environment. The state of California will need to enforce its rights to protect its environmental laws and preserve its air quality management capabilities.

The reduction in federal support for environmental policies is creating a new reality in which states and cities will have to be the primary drivers of action. Cases such as the US Supreme Court’s decision to block the enforcement of EPA’s ‘Good Neighbor Plan’ to reduce NO_X_ emissions from power plants and industrial facilities, which might not have an impact on local O_3_ concentrations but would impact those of neighbouring states [79], could become more common.

Another challenge ahead is the increasingly frequent fires on the outskirts of the city, where development and wildland areas overlap. Aside from the destruction and loss they cause, they release massive amounts of pollutants, including O_3_ precursors, as well as reactive nitrogen and chlorine species, that accelerate O_3_ formation [80]. These fires are triggered by urbanisation trends under a changing climate and a lack of prevention and updated strategies to manage fires. The blazes that devastated the Palisades and Eaton districts in January 2025 are a wake-up call to take immediate action against this threat, which endangers not only the lives of those who live where they spark, but also the lives of all Angelenos due to the significant deterioration in air quality that they cause.

## Beijing–Tianjin–Hebei (*Jing*–*Jin*–*Ji*) region

Over the past decade, China has implemented an impressive suite of policies to clean the air. These policies have been effective in reducing pollutants directly emitted into the atmosphere, such as SO_2_, NO_X_, CO, PM_10_ and PM_2.5_. However, O_3_ pollution remains a challenge in large metropolitan areas, such as in the *Jing***–***Jin***–***Ji* region, although minor reductions have recently been observed since air quality data became available about 10 years ago, presumably due to improved efforts to control both NO_X_ and VOC emissions. Nevertheless, O_3_ concentrations still exceed the Chinese air quality standard for slightly more than 30% of the days, particularly during the summer, resulting in 3.9 to 6.1 premature deaths per 100,000 inhabitants [44]. The reasons preventing significant O_3_ reductions are complex and involve chemical processes that are still not well understood. However, changes in emission patterns and precursor pollutants distribution at regional scale, a changing climate that favours photochemistry, and ironically, larger green areas and decreases in particle pollution all hamper O_3_ reduction efforts.

### General characteristics

Beijing, China’s capital, together with the municipality of Tianjin and the province of Hebei, makes up the BTH, commonly referred to as *Jing*–*Jin–Ji*, an urban agglomeration of 110 million inhabitants that covers 212,000 km^2^ in northeast China.

Beijing is the political, economic, and cultural centre of China. Tianjin is the gateway to northern China, as well as the shipping and logistics centre, and the manufacturing base of the region. Hebei is a major manufacturing province, famous for its steel industry. The *Jing*–*Jin–Ji* region is the largest and most dynamic economy in northern China; its economy has grown 1.8 times in 10 years, reaching a GDP of US$1477 billion (CN¥10,444 billion), equivalent to 8.3% of the national GDP in 2023. However, rapid population growth and massive urban expansion have accompanied this economic growth, altering the landscape, increasing energy consumption and exacerbating traffic congestion and air pollution.

### Ozone trends

**Figure 5:**
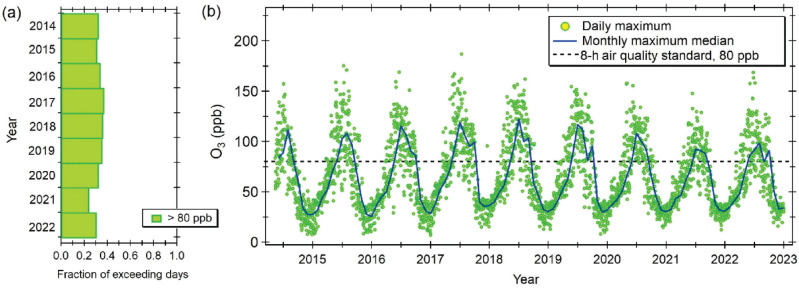
Daily 8-h average maximum O_3_ concentrations. (a) Fraction of days exceeding the current local 8-h air quality standard of 80 ppb. (b) Time series of daily 8-h average maximum concentrations. Green markers indicate the maximum concentration reported by any air quality monitoring station in Beijing and Tianjin. The blue line is the monthly median of the daily maximum concentrations. Data were obtained from the TOAR Database (https://doi.org/10.5281/zenodo.10911196).

China’s air quality nationwide monitoring network was launched in 2013 and has seen no significant change in O_3_ concentrations in the *Jing–Jin–Ji* region, as well as many other highly urbanised areas of the country. Since 2016, the annual 90th percentile of the 8-h average O_3_ concentration has exceeded 85 ppb, reaching 92 ppb in 2023 [81]. Figure 5 shows that O_3_ episodes exceeding the 8-h average threshold of 80 ppb set as air quality standard are unfortunately common; about a third of the days throughout the year exceed this limit. Nonetheless, monthly maximum median concentrations have recently started to decrease, presumably due to improved efforts to control both NO_X_ and VOC emissions [82].

O_3_ concentrations are highest in the summer, driven by fast photochemical production of hydrogen oxide radicals (HO_X_) that can overcome the radical titration caused by high emissions of NO_X_. Under a VOC-limited or NO_X_-saturated regime, as in the urban cores of the *Jing–Jin–Ji* region, the decreasing NO_X_ would increase O_3_ concentrations [83], although in some suburban areas, O_3_ concentrations have started to decrease in response to NO_X_ reductions [84]. Nevertheless, the increased O_3_ trend cannot be simply explained by changes in anthropogenic emissions of NO_X_ and VOCs. Increased urban greenery and changes in particle pollution apparently have a significant impact on O_3_ production.

Along with urban expansion, green spaces have increased 120% in the last two decades, accounting for almost half of the urban area nowadays. This massive expansion of urban vegetation has increased the contribution of biogenic VOCs, enhancing O_3_ formation, especially in the summer. According to numerical simulations, biogenic VOC emissions account for 5–8 ppb of Beijing’s maximum daily 8-h average O_3_ concentration [85]. Similarly, reductions in PM_2.5_ have increased solar radiation reaching the surface, which has increased NO_2_ photolysis rates and slowed down the sink of HO_X_, thus stimulating O_3_ production [83]. Furthermore, the reduction in particle pollution has resulted in changes in aerosol optical properties. Reductions in black carbon have altered the aerosol optical depth and the single-scattering albedo, also accelerating O_3_ production [86]. This suggests that control measures must take into account the combined effect of NO_X_ and VOC emissions in the formation of PM_2.5_ and O_3_.

Regarding the impact of climate change, warmer and drier summers are worsening O_3_ pollution. Longer summers and heat waves have been increasingly common in recent years, bringing with them adverse weather conditions that favour the formation and accumulation of O_3_. Higher temperatures, along with clear skies and stagnation conditions, foster O_3_ production by increasing evaporative and biogenic VOC emissions, as well as VOC reactivity with the hydroxyl radical [87]. Heat wave duration has been found to determine the severity of O_3_ episodes [88].

Zhang et al. [89] found that nitrous acid (HONO) and nitryl chloride (ClNO_2_), two important chemical species in atmospheric chemistry, boost 12–14% O_3_ production in summer. HONO is directly emitted into the air by vehicular traffic and biological activity in agricultural fields, particularly after fertilisation, as well as being formed through heterogeneous chemical processes (i.e., chemical reactions on particles), as also happens with ClNO_2_. The excess of HONO in concert with the high emissions of VOCs seems to be the cause of higher O_3_ concentrations observed in winters in recent years, suggesting a switch to an O_3_ production regime, in which decreasing VOC emissions would avoid further spreading of high O_3_ episodes into the winter and spring [90].

Regional transport also has a role. Studies suggest that polluted plumes from neighbouring provinces, including Shandong, Henan, Jiangsu and Anhui, might account for 35% of the O_3_ concentrations in the *Jing–Jin–Ji* region [91]. The O_3_ production in upper layers within the boundary layer appears to also make a significant contribution. The accumulation of oxygenated VOCs (OVOCs) higher in the atmosphere, supports vigorous O_3_ production, while shifting towards an NO_X_ control regime, as NO_X_ concentrations decline faster than those of OVOCs with increasing height [92]. This indicates that regional O_3_ control strategies must account for vertical variations in O_3_ production and assess the formation regime throughout the entire boundary layer.

### Health and economic impacts

Malashock et al. [4] estimated 2910 premature deaths due to O_3_ exposure for 2019 only for Beijing, while Hu et al. [44] calculated a total toll of 6717 premature deaths for the entire *Jing–Jin–Ji* region for the same year. These authors found a peak of 7391 premature deaths in 2018 when annual concentrations were higher. In 2021 the number of premature deaths fell to 4282, but a slight increase could be expected since then, as annual average concentrations have increased in the last 3 years. Between 2018 and 2021, 3,200,000 to 5,400,000 people each year experienced short-term respiratory and cardiovascular problems as a result of O_3_ exposure. The economic cost imposed by O_3_ pollution in the region fluctuated between US$0.93 and 1.37 billion (CN¥5.96 and 9.06 billion) during that period. According to Wang et al. [93], meeting the long-term targets of current air quality improvement programmes might reduce O_3_-related premature deaths by 89%, preventing an economic loss of US$9.45 billion (CN¥68.4 billion) in 2035 compared to O_3_ levels in 2019.

### Main interventions

Air quality governance in the *Jing–Jin–Ji* region started in the 1990s and focused on pollutants emission control, especially SO_2_ and NO_X_ from coal-burning industries. However, air quality did not improve. Even as emission limits tightened, poor accountability for emissions reporting, as well as a lack of incentives to adopt cleaner technologies, prevented significant improvements [94]. Local administrations were primarily responsible for implementation and supervision, but no standardised methodology and verification mechanisms were in place.

By 2005, the Central Government recognised that improving air quality required more than just reducing emissions from industry, and it expanded control efforts to include other critical pollutants, as well as economic sectors, including energy, transportation and agriculture. The regional nature of air pollution also became clear, and control measures started to be implemented in neighbouring cities and provinces. Beijing’s Olympic Games in 2008 acted as a catalyst for the implementation of regional air quality strategies. Temporal control measures such as halting industrial production, suspending construction, cutting vehicular traffic by more than 50% and significantly reducing other economic activity that could contribute to pollutant emissions proved effective. During the Olympic Games, the local Air Pollution Index was 36% lower than the previous years’ average [95].

In subsequent years, a number of institutional reforms were adopted, as well as surveillance measures implemented to improve air quality management. Air quality targets became mandatory and were integrated into provincial and municipal economic and social development plans. However, such efforts were not sufficient to avoid the air quality crisis of 2013 caused by persistently high, sometimes record-breaking, concentrations of PM_2.5_. The Central Government, in response, enacted the Action Plan on Prevention and Control of Air Pollution the same year, laying down actions to reduce PM_2.5_ concentration by 25% over a 5-year period [96]. Air quality standards were revised, and the air quality topic became relevant in both political discourse and public opinion. Subsequently, the Three-Year Action Plan to Win the Blue-Sky Defense War 2018–2020 was enacted in 2018 [97].

The air pollution control measures included strengthening emission standards for power plants and equipping them with continuous emission monitoring systems, tightening industrial emission standards, phasing out old and inefficient factories, adopting cleaner technologies and installing end-of-pipe pollution control devices in small factories, controlling VOC emissions in the petrochemical industry, eliminating small coal-fired boilers, switching to electricity and gas-powered heating from coal in households, strengthening vehicle emission standards, retiring old vehicles and improving fuel quality [97].

As a result of such control measures, from 2013 to 2017, China reduced its anthropogenic emissions by 59% for SO_2_, 21% for NO_X_, 23% for CO, 36% for PM_10_ and 33% for PM_2.5_. However, VOC emissions increased 2% [98], pointing to a lack of effective measures for VOCs and explaining in part why O_3_ concentrations did not decrease [99].

The current plan underlined in the 14th Five-Year Plan for Ecological Environmental Protection (2021–2025) is the first national plan to mention ground-level O_3_ pollution. It recognises that NO_X_ and VOCs emissions from transportation and industry remain high, and their control is critical to reducing O_3_ pollution. This plan aims at the synergistic control of PM_2.5_ and O_3_, while addressing climate change targets by simultaneously reducing CO_2_ emissions. This plan is based on four principles: (1) science-based pollution control, (2) strengthening legislation, (3) strict standards and (4) increased investment [100].

Importantly, the *Jing–Jin–Ji* region operates under an innovative regional framework designed to coordinate actions between the Central Government and the local governments to prevent and control air pollution [100]. This framework encompasses integrated planning, standardised monitoring, coordinated emergency responses and real-time data sharing across jurisdictions. For such an endeavour, air quality monitoring has been strengthened, and significant investments have been made to build detailed emission inventories and develop air quality forecasting models. Similarly, new mechanisms have been implemented to ensure governance and the enforcement of environmental regulations.

Air quality in the *Jing–Jin–Ji* region has improved. Ambient concentrations of PM_2.5_, as well as other key pollutants, have substantially decreased, proving the effectiveness of the regional framework that has been implemented. However, the region still experiences O_3_ concentrations that exceed recommended guidelines, and high pollution days continue to pose significant health risks. Changes in emission sources of O_3_ precursors and the rapid decrease of PM_2.5_ concentrations, driven by past clean air actions, have influenced O_3_ production [22]. The next phase of air pollution control strategies must simultaneously address air quality standards and climate change targets, specifically focusing on the dual impacts of ambient O_3_ and PM_2.5_ pollution on climate and air quality.

### Challenges ahead

Curbing O_3_ pollution in the *Jing–Jin–Ji* region will require improved VOC emission controls as NO_X_ emissions continue to decrease under current regulations across the three entities. This will require even more stringent environmental policies efficiently coordinated by local, regional and federal agencies. However, some policies in place may appear draconian, at least by Western standards, and the question arises whether implementing even stricter measures could cause social unrest. For example, since 2013, over 3000 manufacturing and polluting enterprises have been closed in the region [101], bringing unemployment and social inequity, and forcing workers to relocate to places far from their hometowns [102].

Similarly, one of the most common wishes among Beijing residents nowadays is to obtain a permit to purchase a car, even though they do not have a pressing need for one [103]. Since 2011, a licence plate lottery system has been used to limit the number of cars that locals can acquire each year in order to reduce traffic congestion and pollutants emission. At the start of this decade, the odds of obtaining a plate were 1 in 2900, and it could take up to 40 years [104]. Initially, people began purchasing cars in neighbouring cities and then bringing them home to evade the lottery system [105]. This loophole was closed by imposing strict driving restrictions on those cars [106]. The lottery system also created a black market for licence plates, where issued plates are resold at higher prices, allowing wealthy residents to circumvent the lottery system [107]. In very recent years, there has been a surge in electric vehicles (EV) that has somewhat alleviated these unforeseen consequences. The price of an EV has dropped since 2023, even though the government ended the subsidy for their purchase that year, at the same time that it launched a subsidy to replace fossil-fuel vehicles with electric ones, and increased their annual quota by making the licence plate lottery system more flexible [108]. Furthermore, EVs are not subject to the same driving restrictions as gasoline cars, allowing drivers to use their vehicles every day. However, this surge of EVs has led to an increase in traffic, extending the hours when Beijing’s roads are gridlocked [109]. This is without taking into account the proliferation of large and heavy EVs that undermines the environmental and economic benefits of the transition towards electric mobility [110].

Air pollution control measures in Beijing must be sustainable, as they should be in any other metropolitan area. The social costs associated with decisive actions following a strict top-down enforcement, as implemented so far in the *Jing–Jin–Ji* region, may prove unsustainable in the long term [111]. Stakeholders must address unforeseen social repercussions and establish information exchange platforms for decision-making and implementation that guarantee social fairness.

## Key learnings

The MAMC, the LA Basin and the *Jing–Jin–Ji* region share the challenge of controlling and reducing ambient O_3_ concentrations. The three metropolitan areas have managed to reduce anthropogenic emissions of primary pollutants, attaining concentrations of regulated pollutants, such as CO, SO_2_, NO_X_ and PM_10_, close to or below air quality standards set to protect public health. However, it is not the case for O_3_ and PM_2.5_, two pollutants whose formation is attributable, as a whole and in part, respectively, to complex chemical reactions in the atmosphere. For the particular case of O_3_, current measures have apparently reached their limit. Stricter emission regulations, improvements in technology and cleaner fuels are no longer sufficient. They have kept O_3_ concentrations at a threshold that, unfortunately, remains high and poses a risk to public health. A different mix of precursor pollutants as a result of the implementation of advanced technology and new regulatory measures, emergent emission sources, and growing urban sprawls with different landscapes under a changing climate have limited their effectiveness and prevented further reductions.

The three metropolitan areas are apparently in a transitional state, moving towards a NO_X_-limited regime, since VOC reductions have been outpaced by NO_X_ reductions. However, the current reductions in NO_X_ have not been sufficient to reduce O_3_ concentrations; on the contrary, they appear to be boosting its production. In the MCMA, a slightly increasing trend in O_3_ concentrations has begun to be recorded. Studies suggest that even more drastic reductions in NO_X_ emissions, larger than those experienced during the COVID-19 pandemic, are needed to reduce ambient O_3_ concentrations under current VOC emissions.

A clear understanding of the chemical reactivity of their atmospheres is necessary to identify the VOCs whose emissions should be controlled in order to contribute to lowering O_3_ formation in concert with NO_X_ reductions. Efforts are needed to address VOC emissions that in the past had been overlooked, such as those related to cooking and so-called VCPs, including evaporative emissions from household cleaning and personal care products. Likewise, biogenic VOC emissions must be properly accounted for, as they are becoming increasingly significant in O_3_ production, especially during the growing season.

Nevertheless, actual ambient O_3_ concentrations cannot be simply explained by changes in anthropogenic and natural emissions of NO_X_ and VOCs. Studies suggest that changes in particle pollution have an impact on O_3_ formation; therefore, control measures must take into account the combined effect of both precursor pollutants in the formation of PM_2.5_ and O_3_. In the same context, meteorological variability triggered by climate change is another factor to consider. Clearer skies, heat waves and wildfires enhance O_3_ formation.

It is also important to consider the expansion of urban sprawls and changes in buildings’ morphology (e.g., increasingly taller buildings), which alter turbulence production, atmospheric stability and wind circulation, affecting ventilation and pollutants dispersion, as well as the spatial and temporal distribution of O_3_ plumes and the location of O_3_ peaks. Finally, as observed for the case of the MCMA and the *Jing–Jin–Ji* region, O_3_ pollution is increasingly becoming a regional issue rather than a local one, while for the case of the LA Basin, hemispheric pollution has a significant impact on background O_3_ concentrations.

## Learned experiences and recommendations

Although the three metropolitan areas examined here have implemented different approaches to tackle O_3_ pollution, there are key learnings that could help other cities to address the problem. However, it is necessary to bear in mind that each city has unique characteristics, and actions implemented in one city may be ineffective or impractical in another. Control measures must be tailored to local conditions, taking into account the climate, geography, topography, social and economic circumstances and even the governance of each city. Yet, the experiences gained while constructing the air quality management systems that have allowed them to understand and monitor their O_3_ problem, along with the subsequent formulation of strategies and implementation of environmental regulations taking into account scientific, operational, administrative, political and social aspects, can be taken as a reference by cities that are struggling to control O_3_ pollution, and in general, improve air quality. In this context, the article concludes with the following set of recommendations to achieve effective and sustained air quality management that contributes to reducing the threat posed by ground-level O_3_. These recommendations draw on the analysis presented here of three iconic metropolitan areas where O_3_ pollution is still a major issue, the authors’ own experience in air quality management [10,11,29,55,112,113], recommendations made by other experts on the subject matter (e.g., Hidy et al. [114]; Pinder et al. [115]; Gani et al. [116]; Sokhi [117]), and guides developed by international organisations such as those prepared by the WHO to assess and address air pollution [118].

- Successful ground-level O_3_ control measures rely on robust and timely air quality management (i.e., air quality monitoring, emission inventories and air quality modelling). It is the basis for taking informed action following a science-policy approach, while also considering the benefits to public health, the local economy and the sustainability and liveability of the city itself. For such an endeavour, specific policy and financial support is required to incentivise air quality management while ensuring it is done correctly through close monitoring of progress and good public governance.
- Effective air quality management depends on reliable air quality monitoring systems at local and regional scales, integrated emission inventories and accurate air quality forecasting models that cover entire metropolitan areas and beyond their borders. Ozone pollution must be seen as a regional challenge to be able to assess the influence that pollution plumes from neighbouring towns and biogenic emissions from nearby rural ecosystems have on the city itself and, in turn, how its emissions and O_3_ plume impact those towns and ecosystems.
- In addition to conventional air quality monitoring stations, it is recommended to have a monitoring supersite. Air quality monitoring supersites are equipped with state-of-the-art instrumentation to provide continuous measurements with higher time resolution, lower detection limits, and a broader range of chemical species and meteorological parameters (e.g., [119,120]). A more comprehensive set of air quality data enables air quality managers to better address the causes of high air pollution levels.
- Scientific research must be at the centre of air quality management. Each one of its components must be supported by scientific research to provide solid foundations for designing effective control measures. Air quality managers must have a thorough understanding of the origin, transformation, dispersion, fate and impact on human health and the ecosystem of O_3_ and its precursor species, as well as other key pollutants.
- Collaborative air quality field campaigns are an effective way to promote scientific research to address air pollution [112]. They involve the deployment of a wide range of regulatory- and research-grade monitors across networks of ground observation sites, tall towers, mobile laboratories and onboard research aircraft. With the assistance of satellite observations and air quality forecasting models, these multidisciplinary studies improve our understanding of the origin, chemistry, dispersion, transport and fate of primary and secondary pollutants, reduce uncertainties in emission estimates and numerical simulations and provide guidance for setting priorities for improving air quality management.
- Although controlling O_3_ pollution in large metropolitan areas should prioritise air quality purposes, air quality and climate change programmes should be coordinated to provide synergistic benefits [121]. For example, actions to reduce emissions of VOCs and NO_X_ from the combustion of fossil fuels contribute to tackling O_3_ pollution, while also cutting CO_2_ emissions. However, this must be done with caution because emission reductions may not always result in lower O_3_ levels.
- States have the responsibility to enact laws and implement regulatory mechanisms that ensure people’s right to breathe clean air with a perspective of human rights and social justice. Under this principle, governments at the local, regional and national levels have to coordinate efforts to improve air quality. Furthermore, governments must develop collaboration mechanisms with other nations to jointly address the problem, recognising that air pollution has no borders.
- Independent initiatives, such as those of non-governmental organisations, that promote public awareness and provide technical information and training are welcome (e.g., [122]). These organisations and the private sector must be integral to efforts to improve air quality, but they must refrain from taking the lead. Air quality management is a permanent and evolving task that can only be undertaken by authorities. Breathing clean air should not be seen as a business opportunity or used to promote partisan ideologies.
- Policymakers must understand the costs and consequences of failing to address the threat posed by ground-level O_3_ and air pollution in general. Under clear transparency rules, together with air quality managers, they must identify financial schemes, including investments in infrastructure and trained personnel, that allow effective and sustained air quality management.
- Scientists and environmental managers must make air quality information more relatable to the public by linking air quality data with reduced air pollution exposure and improved public health [123]. Communication is essential, and it is often the first step in engaging the general public with informed action. The scientific narrative must shift to stories close to people that help them understand the impacts of breathing polluted air. It must connect with people on what they value [124]. Close collaboration with journalists, writers, artists and pedagogues is needed to connect abstract concepts to personal experiences by appealing to people’s emotions. Connecting science with joyful and hopeful emotions is essential to facing environmental challenges.

In closing, it is important to emphasise that ultimately, accurate data, good science and well-chosen technologies can point the way toward corrective regulatory measures, but without strong commitment from government and civil society, no amount of data, science or technology will solve the air pollution problem.

## Data Availability

The datasets generated during and/or analysed during the current study are available from the corresponding author on reasonable request.
